# Etiology and risk factors of stroke in young adults: A multicentric study

**DOI:** 10.1016/j.amsu.2022.104647

**Published:** 2022-09-22

**Authors:** Ayesha Aslam, Ubaid Khan, Farheen Niazi, Iqra Anwar

**Affiliations:** a Department of Neurology, Mayo Hospital, Lahore, Pakistan; b Department of Medicine, King Edward Medical University Lahore, Pakistan; c Pakistan Air Force Hospital, Islamabad, Pakistan; d Mayo Hospital Lahore, Pakistan

**Keywords:** Ischemic stroke, Risk factors, Etiology, Young stroke, Hypertension

## Abstract

**Objective:**

The main objective of this research was to assess the risk factors and causes of ischemic stroke in the young population (age less than 50 years).

**Methods:**

This was a prospective multicenter study conducted at Pakistan Atomic Energy Commission General Hospital, Islamabad, and Mayo hospital Lahore from June 2019 to June 2020. In this research, patients with ischemic stroke, aged 15–50 years were included. Prior to noting demographics, each patient gave ethical approval via filling out consent forms. After that, all demographical details including residence, education, gender and age, and socioeconomic status were noted. Risk factors were evaluated on the questionnaire proforma. Outcomes were measured using the modified Rankin scale (MRS) score. Additionally, data were analyzed by using SPSS V26. A P-value of <0.05 was set as statistically significant.

**Results:**

Out of 80 patients, 53 (66.25%) were male, while 27 (33.75%) were female. Six (7.5%) patients were between the ages of 15 and 25 years, 18 (22.5%) patients were between 26 and 35 years, 48 (60%) patients were between the ages of 36 and 45, and eight (10%) patients were between the ages of 46 and 50. According to this research, hypertension was found to be the most frequently occurring risk factor in 28 participants (35%), Diabetes mellitus in 23 patients (28.75%), dyslipidemia in 20 patients (22.5%), and smoking in 18 patients (22.5%). The etiology remained undetermined in 30 patients (37.5%). Most of the patients (87.5%) reported positive functional outcomes (MRS score 0–2). However, 3 (3.75%) patients died during the study period.

**Conclusion:**

This research showed that common risk factors of ischemic stroke in the local young population included hypertension, diabetes mellitus, and smoking, whereas the etiology of stroke remained unidentified in the majority of patients.

## Highlights

• This study was conducted to evaluate causes and risk factors of ischemic stroke in young population (age less than 50 years).
• This was a prospective multicenter study conducted between June 2019 to June 2020. Patients with ischemic stroke, aged 15–50 years were included.
• Out of 80 patients, Hypertension was the most frequently found risk factor in 28 patients (35%), followed by Diabetes mellitus in 23 patients (28.75%), dyslipidemia in 20 patients (22.5%), and smoking in 18 patients (22.5%). The etiology remained undetermined in 30 patients (37.5%).
• This research showed that common risk factors of ischemic stroke in the local young population included hypertension, diabetes mellitus and smoking.

## 1 Introduction

Stroke is the second most frequently occurring cause of mortality and the third most commonly found cause of disability globally [1]. Rendering to the 2010 Global Burden of Disease Study, in southeast Asia, Stroke is the most common cause of disability-adjusted life years (DALYs) [2]. Moreover, stroke is more prevalent in the 15- 50-year-old age group in low-income countries and accounts for higher mortality compared to high-income countries [3].Annually, over 800,000 individuals suffer from stroke. Stroke incidence is declining in the West but is potentially high in Asia. However, the mortality rate due to stroke is increasing worldwide [3].Additionally, stroke has a significant impact on the national economy in terms of long-term care and rehabilitation [2].

There are many risk factors and etiologies known to be linked with the occurrence of Ischemic stroke, however, there remains a significant percentage in the younger age group with undetermined causes. For example, previous studies conducted in Europe and the United States indicate that ischemic stroke is increasing in younger adults and traditional stroke risk factors common in older adults (obesity, hypertension, diabetes mellitus, dyslipidemia, and tobacco use) are also common in young acute stroke patients [[4], [5], [6], [7]]. Recent evidence also suggests that the prevalence of conventional risk factors is also considerably high in the 15–55 years old age group compared to the older age group [7]. These reports are primarily from cohorts from North America and Western Europe; however, data on the prevalence of stroke in young patients from Asia and Eastern Europe is insufficient.

Moreover, numerous researches reported an increase in the occurrence of ischemic stroke in the age group between 30 and 45 years [8,9]. Although epidemiological research conducted on the Pakistani population is scarce, only a few hospital-based studies have indicated a higher rate of young stroke in the Pakistani population. A clinical study performed by Khan JA et al. reported incidence of stroke was higher in patients (26%) between 15 and 45 years of age [10]. Furthermore, a case series study by Vohra et al. reported that 34% of their stroke patients were under the age of 50 years [11].

Additionally, evidence suggests developing countries bear over two-thirds of the worldwide burden of stroke [12]. To date, in India, young individuals account for 10%–30% of all stroke cases, compared to 3% to 8.5% in Western countries [[13], [14], [15]] Similarly, the incidence of stroke is also high among young individuals in Pakistan; it may potentially lead to raised economic burden. Moreover, evidence suggests that hypertension was found to be the most frequently observed risk factor in young patients [16,17]. Additionally, etiological subtyping differs based on regional distribution. Thus, it is crucial to evaluate the risk factors and causes of stroke in young patients to prevent disability and stroke reappearances among the younger population.^17^This study is in accordance with strocss guidelines [18].

The aim of this research was to evaluate the risk factors and causes of ischemic stroke in the young population of Pakistan which hasn't been exclusively studied in the past.

**Objectives:** To identify the risk factors and etiology of stroke in young adults less than the age of 50 years.

## 2 Methods

**Study design and Study area:** This study was conducted at the Department of Neurology, Pakistan Atomic Energy Commission General Hospital, Islamabad, and Mayo hospital Lahore from June 2019 to June 2020. Patients below 15 years and those with intracerebral hemorrhage, brain injury, venous sinus thrombosis, head trauma above 50 years of age, and previous history of stroke were excluded from this study. This study included 80 patients of both sexes between the ages of 15 and 50 who presented with their very first episode of ischemic stroke. Data were collected from medical records of patients, laboratory investigations, diagnostic tests, radiological images, and reports. After ethical approval and informed consent from patients, comprehensive demographic details including age, residence, socioeconomic status, and sex were recorded on the proforma. “Complete blood count test” (CBC), “Erythrocyte sedimentation rate” (ESR), glycosylated hemoglobin urine test (Hb A1C), “liver function test”, “renal functional tests” and lipid profile were done for all patients at baseline. Patients with indications underwent immunologic investigations (anti-ds DNA and anti-nuclear antibodies) and thrombophilia screening. Echocardiogram, carotid Doppler, and 24 h ECG recording were also undertaken. CT scan brain was performed on all patients at admission. MRI brain with stroke protocol was also done in cases where needed. Dyslipidemia was defined as having total cholesterol over 240 mg/dL and LDL cholesterol over 160 mg/dL while taking a lipid-lowering agent. Additionally, diabetes mellitus was defined as being treated with insulin or oral hypoglycemic agents and having a glycosylated hemoglobin level of less than 6.5%. TOAST criteria were used to classify stroke subtypes (“Trial of Org 10172 in Acute Stroke Treatment”). Consideration was given to causes of ischemic stroke. Risk factors were recorded. The outcome measures were evaluated using a modified “Rankin scale score”. The mortality rate was also observed. SPSS V25 was used to examine all of the statistical data. The Chi-square test was applied to check the differences between categorical variables. All variables' percentage, mean, and standard deviation were calculated statistically. P-value of <0.05 was accepted as statistically significant. Work has been reported in line with the STROCSS criteria [18].

## 3 Results

Out of 80 patients with Ischemic stroke, 53 (66.25%) were found to be male while 27 (33.75%) patients were females. A total of 6 (7.5%) patients were from 15 to 25 years old, 18 (22.5%) from 26 to 35 years old, 48 (60%) from 36 to 45 years old and 8 (10%) were from 46 to 50 years. In this research 45 (56.25%) participants were from rural areas, whereas 35 (43.75%) were from large cities. In addition, 42 (52.5%) patients had a low socioeconomic status, whereas 38 (47.5%) had an intermediate socioeconomic status (Table 1).

**Table 1:** Baseline characteristics of all the patients.

Characteristics	Frequency
**Gender**	
Male	66.25% (n = 53)
Female	33.75% (n = 27)
**Age (years)**	
15 to 25	7.5%(n = 6)
26 to 35	22.5%(n = 18)
36 to 45	60%(n = 48)
Above 45	10%(n = 8)
**Residence**	
Urban	56.25%(n = 45)
Rural	43.75%(n = 35)
**Socio-Eco Status**	
Low	52.5%(n = 42)
Middle	47.5%(n = 38)

**Fig. 1:**
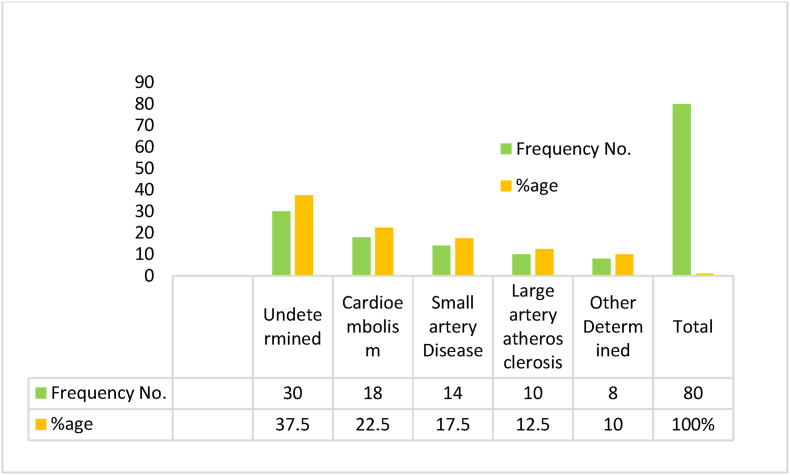
Etiology of Ischemic Stroke among all the patients.

In thirty patients, or 77.5%, the most common reason for the ischemic stroke was unknown. Additionally, in 18 (22.5%) patients cardio embolism was found to be a major etiology for the development of stroke. Moreover, in 14 (17.5%), small artery disease was observed which led to the occurrence of stroke. Other causes of stroke included “large artery atherosclerosis” observed in 10 (12.5%), while other 8 (10%) patients experienced stroke due to unknown causes (Fig. 1).

Hypertension was the most common risk factor, accounting for 35% of cases, followed by dyslipidemia (25%), diabetes mellitus (23%), vasculitis in 6% of cases

smoking (25%), “coronary artery disease” in 4% of cases and, atrial fibrillation in 7% of cases. (Table 2).

**Table 2:** Distribution of risk factors of stroke among all the patients according to the gender.

Risk Factors	Male	Female	Total (%)	P-value
	n = 53 (66.25%)	n = 27 (33.75%)	n = 80	<0.05
Hypertension	22	6	28 (35%)	**<0.05**
	(41.51%)	(22.22%)		
Diabetes	17	6 (22.22%)	23 (28.75%)	**<0.05**
	(32.08%)			
Dyslipidemia	15	5	20 (25%)	**<0.05**
	(26.32%)	(18.52%)		
Smoking	15	3	18 (22.5%)	**<0.05**
	(26.32%)	(11.11%)		
Vasculitis	0 (0%)	5	5 (6.25%)	
		(18.52%)		
AF	4	2	6 (7.5%)	**<0.05**
	(7.55%)	(7.41%)		
Coronary artery Disease	2	2	4 (5%)	**<0.05**
	(3.77%)	(7.41%)		

The majority of patients, 60 (75%), had two or more risk factors, with the exception of six patients who only had atrial fibrillation and two patients who only had coronary artery syndrome as risk factors. Vasculitis was found a risk factor in 18.52% of the female subgroup.

In this research, outcome measures were noted according to the “modified Rankin scale scoring system” and it was observed that 70 (87.5%) patients scored 0 to 2, 5 (6.25%) scored 3–4 and the remaining 5 (6.25%) scored 5–6 (Table 3).

**Table 3:** Outcome measures at the time of Discharge (“modified Rankin Scale Score”).

MRS Score	Frequency No.	%age
0–2	70	87.5
3–4	5	6.25
5–6	5	6.25
**Mortality**		
Yes	3	3.75
No	77	96.25

## 4 Discussion

Ischemic stroke is one of the most frequently occurring neurological disorders with significant mortality and morbidity rates globally. Numerous studies have investigated the risk factors and causes associated with ischemic strokes in young populations [14,15]. However, this is the first study that has investigated the risk factors and causes of stroke collectively in the Pakistani Population. The main aim of this research was to investigate the risk factors and specific etiologies of strokes in the young adult Pakistani population. In our study, 66.25% of stroke patients were males whereas 37.75% of stroke patients were females. These results are similar to those of previous research in which the percentage of male patients who experienced stroke was 60–70% compared to 30% of female patients ages [16,17]. In our study, we observed that 82.5% of patients were between the ages of 26 and 45 and 60% of patients were between the ages of 36 and 45. These results demonstrated that male patients between the ages of 36 and 45 are particularly susceptible to developing stroke. Numerous researches have also demonstrated similar findings identical to ours, in which the majority of stroke patients were of the ages 30–45 years [19,20].

In this research, 52.5% of patients had a low socioeconomic status, whereas 47.5% had an intermediate socioeconomic status. These outcomes were identical to those of other trials in which the majority of young patients had a poor socioeconomic status [21].

This implies that male gender and poor socioeconomic status could be a risk factors for the occurrence of stroke in young individuals. Additionally, in this investigation, the leading cause of ischemic strokes was unknown (77.5%), followed by cardioembolism the secondary major cause (22.5%). Moreover, Khalid Sher et al., found that 25.3% of deaths in young patients were due to cardioembolism, which was the top cause of mortality [22]. Similarly, according to a study by Deepa D et al., an undetermined cause was found to be the most frequently occurring cause of ischemic strokes in a young population [23].

In this research the most 35% of patients had hypertension, which was the most prevalent risk factor detected, followed by diabetes mellitus (28.75%), dyslipidemia (25%), smoking (22.5%), atrial fibrillation in 6 (7.5%), and coronary artery disease (5%) individuals. These results are identical to another research, Schneider et al. observed 741 cases and found that hypertension was the most common risk factor among young adults. Dyslipidemia (46%) and smoking were the next most common risk factors (35%)[24].Numerous additional studies reported risk factors for ischemic strokes that were similar to ours, with dyslipidemia, smoking, diabetes, and hypertension being the most prevalent in the stroke population of young patients [25,26]. Similarly, our research was supported by another medical research, in which the most common risk factor was being between 36 and 45 years old. Smoking, hypertension and diabetes were the next most commonly reported causes [27].

In this research, outcomes were measured using the “modified Rankin scale scoring system’’, and it was observed that 87.5% of patients had scores between 0 and 2,5 whereas 6.2% of patients had scores between 3 and 4, and 6.2% had scores between 5 and 6. These results are similar to a previous trial in which 89% of participants had favorable outcomes with a relatively low death rate [28].

Our study has a few limitations: it included small sample size and further extensive investigations could not be carried out in patients with undetermined etiology due to limited resources.

## 5 Conclusion

The study concludes that male patients aged 36–45 years were at higher risk of developing stroke and hypertension was found to be the most commonly found risk factor subsequent to smoking, dyslipidemia, and Diabetes. The cause of stroke in the young population still remains undetermined in the majority of patients. Our study findings emphasize the importance of proactive care of conventional risk factors and comprehensive patient work-up to determine the cause of stroke in young people of Pakistan. Additionally, further longitudinal studies on a larger scale on young stroke are required to understand the potential reasons and risk factors behind stroke development in young patients.

## Ethical approval

This cross-sectional study was approved by the Institutional Review Board by PAEC General Hospital Islamabad.

{PGHI-IRB(Me)-RCD-06-001)}

## Sources funding

There is no source of funding.

## Author contribution

All authors have contributed equally.

## Trial register number

N/A.

## Registration of Research Studies

N/A.

## Guarantor

Dr Ayesha Aslam is guarantor.

## Consent

N/A.

## Declaration of competing interest

The authors declare that they have no known competing financial interests or personal relationships that could have appeared to influence the work reported in this paper.
