# Supplementary material for: Etiology and risk factors of stroke in young adults: A multicentric study
Source: Ann Med Surg (Lond). 2022 Sep 22;82:104647. doi: 10.1016/j.amsu.2022.104647 (PMC9577644; doi:10.1016/j.amsu.2022.104647)
Supplement: Multimedia component 1 [file mmc1.docx]

| The STROCSS 2021 Guideline | | | |
| --- | --- | --- | --- |
| Item no. | **Item description** | **Page** |  |
| TITLE | | | |
| 1 | **Title**  Etiology and risk factors of stroke in young adults: A cross-sectional prospective multicentre study. |  |  |
| ABSTRACT | | | |
| 2a | **Introduction**   - Stroke is the second most frequently occurring cause of mortality and the third most commonly found cause of disability globally. - The etiology of stroke differs based on regional distribution and age. Therefore, it is crucial to identify the risk factors and causes of stroke in young patients to prevent disability and stroke recurrences among the younger population - This study was conducted to evaluate the causes and risk factors of ischemic stroke in a young population (age less than 50 years). |  |  |
| 2b | **Methods**   - A cross-sectional prospective multicentre study was conducted from June 2019 to June 2020 on 80 patients of both genders with ages between 15 to 50 years. - The primary outcome measure was disability. There was no secondary outcome measure. |  |  |
| 2c | **Results**   - Hypertension was the most frequently found risk factor in 28 patients (35%), followed by Diabetes mellitus in 23 patients (28.75%), dyslipidemia in 20 patients (22.5%), and smoking in 18 patients (22.5%). The etiology remained undetermined in 30 patients (37.5%). The majority of the patients (87.5%) had good functional outcomes (mRs score 0-2). |  |  |
| 2d | **Conclusion**   - This study concluded that male patients aged 36 to 45 years were at higher risk of developing stroke and hypertension was the most commonly found risk factor subsequent to Diabetes, dyslipidemia, and smoking whereas the etiology of stroke remained unidentified in the majority of patients. - Our study sample size was small, future studies on large sample sizes are required to understand the etiology and risk factors of stroke. Additionally, there was a limitation of sources. - With the help of this study, better treatment techniques focusing on the specific mechanism and risk factors that causes stroke may be developed. |  |  |
| INTRODUCTION | | | |
| 3 | **Introduction**   - Stroke is the second most frequently occurring cause of mortality and the third most commonly found cause of disability globally1. - Recent evidence suggests that the prevalence of conventional risk factors is also considerably high in the 15 to 55 years old age group compared to the older age group.7 These reports are primarily from cohorts from North America and Western Europe; however, data on the prevalence of stroke in young patients from Eastern Europe and Asia has been lacking, only a few Pakistani hospital-based studies have indicated a higher rate of young stroke in the population. - The objective of this study is to identify the risk factors and etiology of stroke in young adults less than the age of 50 years. |  |  |
| METHODS | | | |
| 4a | **Registration**   - *N/A* |  |  |
| 4b | **Ethical approval**   - Ethical approval of a research study contributes to the legitimacy of the research findings. This is critical for people who will make decisions based on the research findings - All 80 patients gave ethical approval for the study |  |  |
| 4c | **Protocol**   - N/A |  |  |
| 4d | **Patient and public involvement in research**   - N/A |  |  |
| 5a | **Study design**   - A cross-sectional prospective multicentred study. |  |  |
| 5b | **Setting and timeframe of research**   - This study was conducted at the Department of Neurology, Pakistan Atomic Energy Commission General Hospital, Islamabad, and Mayo hospital Lahore from June 2019 to June 2020 |  |  |
| 5c | **Study groups** |  |  |
| 5d | **Subgroup analysis**   - Complete blood count test (CBC), Erythrocyte sedimentation rate (ESR), urine test, liver function test, glycosylated hemoglobin (Hb A1C), lipid profile, and renal function tests were done for all patients at baseline. Thrombophilia screening and immunologic studies (anti-nuclear and anti-ds DNA antibodies) were performed in patients when indicated. Echocardiogram, carotid Doppler 24 hours ECG recording, CT scan brain, and MRI brain were performed. |  |  |
| 6a | **Participants**   - with first-ever ischemic stroke were included in this study. Patients below 15 years and those with head trauma, brain injury, intracerebral hemorrhage, venous sinus thrombosis, above 50 years of age, and previous history of stroke were excluded from this study. |  |  |
| 6b | **Recruitment**   - Written consents were taken from patients. |  |  |
| 6c | **Sample size** – comprehensively describe:   - The sample size was calculated using the Raosoft online sample size calculator.   The confidence interval was 95%   - The margin of error was 5% |  |  |
| METHODS - INTERVENTION, AND CONSIDERATIONS | | | |
| 7a | **Pre-intervention considerations** – comprehensively describe:   - Preoperative patient optimization (e.g. weight loss, smoking cessation, glycaemic control, etc.) - Pre-intervention treatment (e.g. medication review, bowel preparation, correcting hypothermia/-volemia/-tension, mitigating bleeding risk, ICU care, etc.) |  |  |
| 7b | **Intervention** – comprehensively describe:   - Type of intervention and reasoning (e.g. pharmacological, surgical, physiotherapy, psychological, etc.) - Aim of intervention (preventative/therapeutic) - Concurrent treatments (e.g. antibiotics, analgesia, anti-emetics, VTE prophylaxis, etc.) - Manufacturer and model details, where applicable |  |  |
| 7c | **Intra-intervention considerations** – comprehensively describe:   - Details pertaining to the administration of intervention (e.g. anesthetic, positioning, location, preparation, equipment needed, devices, sutures, operative techniques, operative time, etc.) - Details of pharmacological therapies used, including formulation, dosages, routes, and durations - Figures and other media are used to illustrate |  |  |
| 7d | **Operator details** – comprehensively describe:   - Requirement for additional training - The learning curve for technique - Relevant training, specialization, and operator's experience (e.g. average number of the relevant procedures performed annually) |  |  |
| 7e | **Quality control** – comprehensively describe:   - Measures are taken to reduce inter-operator variability - Measures are taken to ensure consistency in other aspects of intervention delivery - Measures are taken to ensure quality in intervention delivery |  |  |
| 7f | **Post-intervention considerations** – comprehensively describe:   - Post-operative instructions (e.g. avoid heavy lifting) and care - Follow-up measures - Future surveillance requirements (e.g. blood tests, imaging, etc.) |  |  |
| 8 | **Outcomes** – comprehensively describe:   - Primary outcomes, including validation, where applicable - Secondary outcomes, where appropriate - Definition of outcomes - If any validated outcome measurement tools are used, give a full reference - The follow-up period for outcome assessment, divided by group |  |  |
| 9 | **Statistics** –   - SPSS V26 was used to analyse the data. - All variables' percentage, mean, and standard deviation were calculated statistically. Analysis approach (e.g. intention to treat/per protocol) - The Chi-square test was applied to check the differences between categorical variables - A P-value of <0.05 was set as statistically significant. |  |  |
| RESULTS | | | |
| 10a | **Participants**   - This study included a total of 80 patients 53 males and 27 females aged between 15 to 50 years. - . |  |  |
| 10b | **Participant comparison**   - Out of 80 patients, 53 (66.25%) were male, while 27 (33.75%) were female. Six (7.5%) patients were between the ages of 15 and 25yrs, 18 (22.5%) patients were between 26 and 35 years, 48 (60%) patients were between the ages of 36 and 45, and eight (10%) patients were between the ages of 46 and 50.   Baseline characteristics of patients   \| **Characteristics** \| **Frequency** \| \| --- \| --- \| \| **Gender** \|  \| \| Male \| 66.25% (n=53) \| \| Female \| 33.75% (n=27) \| \| **Age (years)** \|  \| \| 15 to 25 \| 7.5%(n=6) \| \| 26 to 35 \| 22.5%(n=18) \| \| 36 to 45 \| 60%(n=48) \| \| Above 45 \| 10%(n=8) \| \| **Residence** \|  \| \| Urban \| 56.25%(n=45) \| \| Rural \| 43.75%(n=35) \| \| **Socio-Eco Status** \|  \| \| Low \| 52.5%(n=42) \| \| Middle \| 47.5%(n=38) \| |  |  |
| 10c | **Intervention** – comprehensively describe:   - Degree of the novelty of intervention - Learning required for interventions - Any changes to interventions, with rationale and diagram, if appropriate |  |  |
| 11a | **Outcomes**   - 70 (87.5%) patients scored 0 to 2, 5 (6.25%) scored 3-4 and the remaining 5 (6.25%) scored 5-6 |  |  |
| 11b | **Tolerance** – comprehensively describe:   - Assessment of tolerability of exposure/intervention - Cross-over with explanation - Loss to follow-up (fraction and percentage), with reasons |  |  |
| 11c | **Complications** – comprehensively describe:   - No complications were reported. However, three patients died at the time of discharge. |  |  |
| 12 | **Key results**  The most common etiology of ischemic stroke was undetermined 30 (77.5%), followed by cardioembolism 18 (22.5%), small artery disease found in 14 (17.5%) patients, large artery atherosclerosis in 10 (12.5%) patients and 8 (10%) patients had other determining causes (Figure 1).  The most common risk factor was hypertension 28 (35%) followed by diabetes mellitus 23 (28.75%), dyslipidemia 20 (25%), smoking 18 (22.5%), vasculitis in 5 (6.25%) patients, atrial fibrillation found in 6 (7.5%) and coronary artery disease found in 4 (5%) patients respectively. (Table 2).  Most of the patients 60 (75%) had two or more risk factors except six patients with atrial fibrillation and two with coronary artery syndrome which was the only risk factors. Vasculitides were found a risk factor in 18.52% of the female subgroup.  Outcomes at the time of Discharge (modified Rankin Scale Score)   \| **MRS Score** \| **Frequency No.** \| **%age** \| \| --- \| --- \| --- \| \| 0-2 \| 70 \| 87.5 \| \| 3-4 \| 5 \| 6.25 \| \| 5-6 \| 5 \| 6.25 \| \| **Mortality** \|  \|  \| \| Yes \| 3 \| 3.75 \| \| No \| 77 \| 96.25 \|   Gender wise distribution of risk factors among all the patients   \| **Risk Factors** \| **Male** \| **Female** \| **Total (%)** \| **P-value** \| \| --- \| --- \| --- \| --- \| --- \| \|  \| **n=53**  **(66.25%)** \| **n=27**  **(33.75%)** \| **n=80** \| **<0.05** \| \| Hypertension \| 22  (41.51%) \| 6  (22.22%) \| 28 (35%) \| **<0.05** \| \| Diabetes \| 17  (32.08%) \| 6 (22.22%) \| 23 (28.75%) \| **<0.05** \| \| Dyslipidemia \| 15  (26.32%) \| 5  (18.52%) \| 20 (25%) \| **<0.05** \| \| Smoking \| 15  (26.32%) \| 3  (11.11%) \| 18 (22.5%) \| **<0.05** \| \| Vasculitis \| 0 (0%) \| 5  (18.52%) \| 5 (6.25%) \|  \| \| AF \| 4  (7.55%) \| 2  (7.41%) \| 6 (7.5%) \| **<0.05** \| \| Coronary artery Disease \| 2  (3.77%) \| 2  (7.41%) \| 4 (5%) \| **<0.05** \| \|  \|  \|  \|  \|  \| |  |  |
| DISCUSSION | | | |
| 13 | **Discussion**   - The study concludes that male patients aged 36 to 45 years were at higher risk of developing stroke and hypertension was the most commonly found risk factor subsequent to Diabetes, dyslipidemia, and smoking. The cause of stroke in the young population still remains undetermined in the majority of patients. - Identification of risk factors in young patients belonging to different areas could lead to better risk factor identification as well as a stepping stone toward a better understanding of disease mechanisms. In Pakistan, the causes and Etiology of stroke among younger individuals have not been exclusively studied in the past. - Our study findings emphasize the importance of proactive care of conventional risk factors and comprehensive patient work-up to determine the cause of stroke in young people of Pakistan. |  |  |
| 14 | **Strengths and limitations**   - Our study findings emphasize the importance of proactive care of conventional risk factors and comprehensive patient work-up to determine the cause of stroke in young people of Pakistan. - Our study sample size was small. Additionally, there was a limitation of sources |  |  |
| 15 | **Relevance and implications**   - With the help of this study, better treatment techniques focusing on the specific mechanism and risk factors that causes stroke may be developed. - Future longitudinal studies on a large sample size are required to understand the Etiology and risk factors of stroke. |  |  |
| CONCLUSION | | | |
| 16 | **Conclusions**   - The study concludes that male patients aged 36 to 45 years were at higher risk of developing stroke and hypertension was the most commonly found risk factor subsequent to Diabetes, dyslipidemia, and smoking. The cause of stroke in the young population still remains undetermined in the majority of patients. - Future studies should focus on a large sample size and TOAST subtyping criteria should be modified. |  |  |
| DECLARATIONS | | | |
| 17a | **Conflicts of interest**   - None to declare. |  |  |
| 17b | **Funding**   - Sources of funding (e.g. grant details), if any, are clearly stated - Role of funder |  |  |
| 17c | **Contributorship**   - Acknowledge patient and public involvement in research; report the extent of involvement of each contributor |  |  |
